# The influence of family support during endoscopic submucosal dissection on patient's anxiety

**DOI:** 10.3389/fpubh.2022.992018

**Published:** 2022-10-26

**Authors:** Ruo-Yu Gao, Ri-Yun Gan, Jia-Lan Huang, Ting-Ting Liu, Ben-Hua Wu, Li-Sheng Wang, De-Feng Li, Jun Yao

**Affiliations:** ^1^The Second Clinical Medical College, Jinan University, Shenzhen, China; ^2^Department of Gastroenterology, Shenzhen Luohu People's Hospital, Shenzhen, China; ^3^Department of Gastroenterology, Shenzhen People's Hospital, Second Clinical Medical College of Jinan University, Shenzhen, China

**Keywords:** depression, anxiety, endoscopic submucosal dissection (ESD), complications, family

## Abstract

**Background:**

Psychological problems may promote peptic ulcers. Ulcer-like wounds can be formed after gastric endoscopic submucosal dissection (ESD). The influence of family support on the healing of gastric ESD-induced ulcers remains largely undetermined.

**Objective:**

In the present study, we aimed to assess the Hospital Anxiety and Depression Scale (HADS) scores and the incidence of post-ESD complications in patients with family support in the care process and those in the non-relative group.

**Materials and methods:**

A total of 191 patients aged between 30 and 70 years who received gastric ESD were evaluated with the Chinese version of HADS. Differences in depression and anxiety between the two groups were compared using the chi-square test and *t*-test. Multivariable logistic regression models were used to examine whether anxiety and depression were the risk factors for post-ESD complications.

**Results:**

The mean values of HADS-A (4.61 ± 2.89 vs. 5.56 ± 3.07, *p* = 0.042) and HADS-D (4.14 ± 3.03 vs. 4.97 ± 2.61, *p* = 0.048) scores were significantly lower in patients with accompanying relatives compared with those in the non-relative group. Besides, through the pre-ESD and post-ESD self-contrast, the scores of anxiety and depression in the relative-group were 0.57 and 0.56, respectively (*p* < 0.001), while those in the non-relative group were increased by 1.43 and 1.49, respectively (*p* < 0.001). Multivariable logistic regression analysis revealed that HADS-A, HADS-D scores, and age were significantly correlated with post-ESD abdominal pain (*P* < 0.05).

**Conclusions:**

The occurrence and degree of adverse emotions such as psychological anxiety and depression in patients who received gastric ESD with accompanying relatives during hospitalization may were reduced, and the incidence of gastric post-ESD abdominal pain may was also decreased.

## Introduction

Upper gastrointestinal cancers are the most common leading causes of cancer mortality worldwide (1), accounting for 13.7% of all cancer-related deaths (2). Every year, about 1.5 million people are diagnosed with gastric or esophageal cancers (3, 4) posing tremendous challenges to the healthcare system due to their aggressive presentation (5). As a new minimally invasive technique, endoscopic mucosal dissection (ESD) is used to treat gastrointestinal (GI) superficial neoplasias (6, 7). ESD is a technically complex process, and it removes a large area of the mucosa that may increase the risk of adverse events, such as pain, bleeding, and perforation (8–10). Delayed bleeding is the most important adverse event associated with ESD (10). Since patients may have fear of the operation, apprehension about their illness, and the ESD may cause some pain and discomfort, they are prone to psychological anxiety and depression during the perioperative period.

Health anxiety or depression is a common problem in the community (11), which imposes a huge burden on health services (12). Studies have shown that certain inflammatory diseases are associated with bad mood. Inflammation caused by anxiety and depression is the most common reason for GI mucosal injury (13). Its damage to the GI mucosa may involve a variety of different psychophysiological mechanisms, from stress stimulation of thyroid-stimulating hormone (TSH, a peptic ulcer promoter) (14) to local blood flow changes (15), leading to damage to the gastric mucosal barrier.

Peptic ulcer belongs to the category of typical psychosomatic diseases, and psycho-social factors play an important role in its pathogenesis (16). In recent years, psychological intervention can significantly reduce the degree of anxiety and depression, resulting in enhanced quality of life of patients (17). Family support is one of the important ways of psychological intervention, which can bring mental security to patients. Therefore, the production of negative emotions can be reduced accordingly, and family support plays an important role in the healing of GI mucosal injury (18, 19).

Patients planning to receive gastric ESD may experience psychological distress. We conducted a literature search of PubMed, searching the years 1990–2022,no study has evaluated the impacts of relatives on anxiety, depression, and complications in patients receiving gastric ESD. In the present study, we aimed to assess the prevalence of anxiety, depression, and ESD complications in patients.

## Patients and methods

### Patients

**Figure 1:**
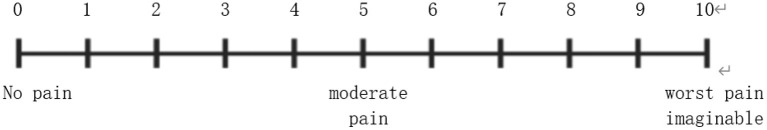
Visual analog scale for pain.

A total of 220 patients who underwent their first gastric ESD at the Shenzhen People's Hospital from January 2021 to May 2021 were enrolled in this study. The patients were divided into the relative group and non-relative group according to whether they were accompanied by relatives or not during the perioperative period. During the perioperative period, patients looked after by relatives were set up as the relative group (*n* = 92), and those looked after by non-relatives (hired caregivers) were set up as the non-relative group (*n* = 89). After the detailed screening, 29 patients were excluded from this study. The procedure and results of screening, as well as the patient classification, were shown in the flowchart (Figure 1). In the relative group, there were 43 males and 49 females aged 30–70, with an average age of 51.9 ± 9.1 years. In terms of disease type, there were four cases of high-grade intraepithelial neoplasia (HGIN**)**, 74 cases of low-grade intraepithelial neoplasia (LGIN), one case of atrophic gastritis, three cases of superficial gastritis, and 10 cases of raised lesions. In the non-relative group, there were 48 males and 41 females aged 30–69, with an average age of 50.1 ± 7.5 years. In terms of disease type, there were three cases of HGIN, 73 cases of LGIN, four cases of superficial gastritis, and nine cases of raised lesions. The inclusion criteria were set as follows: (1) 18–80 years old; (2) diagnosed with early GI tumors or raised lesions; and (3) receiving ESD treatment and willing to provide informed consent. The exclusion criteria were as follows: (1) patients with severe systemic diseases, including kidney, liver, or heart dysfunction; (2) patients with a previous history of anxiety/depression or admission anxiety/depression score ≥8; and (3) patients with Mallory-Weiss syndrome, post-gastrectomy, and coagulation dysfunction. The general data on age, sex, and type of disease between the two groups were not significantly different (*p* > 0.05; **Table 2**). The study was approved by the Ethics Committee of Shenzhen people's Hospital (approval No. of the ethic committee: KY-LL−2020114-01) and registered at ClinicalTrials.gov with the identifier ChiCTR2000032851.

### Methods

On the following day of admission, all patients were informed of the purpose and procedures of the study. If patients were willing to participate, they were asked to sign an informed consent form. Trained researchers recorded demographic and baseline clinical characteristics of each patient and then assisted the patients to perform the test using the Chinese version of the HADS. Additionally, in the family section of the survey, basic demographic information was collected on the closest family member who accompanied the patient more in the course of hospitalization. Both groups of patients underwent gastric ESD. The patients in the non-relative group were given the routine procedure as follows: the doctor introduced the surgical method to the patient and relieved the patient's tension before the operation. Postoperative proton-pump inhibitor (PPI) therapy can promote the healing of ESD-induced ulcers and reduce the risk of bleeding and abdominal pain. For the relative-group, besides the above-mentioned routine procedures, the accompanying family members were informed to communicate more with the patient, patiently listen to the patient's ideas and concerns, encourage the patient to relax, and provide the patient with a warm, quiet, and comfortable environment. On the first day after operation, the researchers recorded the HADS score and postoperative complications of both groups. Abdominal pain after the ESD procedure was assessed by visual analog scale (VAS) (Figure 1). The VAS consists of a 10-cm long horizontal line with its extremes marked as “no pain” and “worst pain imaginable.” Each patient ticked her pain level on the line, and this self-report of pain is considered as the gold standard for pain measurement (20). It was considered that VAS score ≥3 was positive for postoperative abdominal pain. For the evaluation of bleeding after ESD, we think that the patients' gastric drainage tube continuously drains bright red fluid, which is ineffective after conservative drug treatment and needs further endoscopic hemostatic treatment.

### Measures

The severity of the patient's anxiety and depression was scored with the Hospital Anxiety and Depression Scale (HADS) (21), which is a commonly used self-assessment scale to assess the psychological distress in non-psychiatric patients. The HADS questionnaire has been translated into many languages and applied in different countries and regions (22, 23). The rating of the HADS scale is shown in Table 1 (24). HADS is a self-report questionnaire consisting of 14 items, including seven items assessing anxiety (HADS-A) and the other seven items assessing depression (HADS-D). The total score of each subscale obtained ranges from 0 to 21 (higher scores indicate higher anxiety/depression level). In the present study, the demarcation point of 8 was used to diagnose anxiety and depression (22, 25). The HADS-A1 and HADS-D1 were defined as the anxiety and depression subscales of HADS for the relative group, respectively. HADS-A2 and HADS-D2 were defined as the anxiety and depression subscales of HADS for the non-relative group, respectively.

**Table 1:** HADS score.

**For both scales, scores of less than 7 indicate non-cases**	
8–10	Mild
11–14	Moderate
15–21	Severe

### Statistical analysis

All data were statistically analyzed using R software (Version 3.5.3). Continuous variables were expressed as means and standard deviations (SD) and compared using the *t*-test. Categorical data were expressed as percentages and compared using the chi-square test. Normal distribution was assessed by the Kolmogorov-Smirnov test. The independent-samples *t*-test was used for normally distributed continuous variables. and the Mann-Whitney *U*-test was used for non-normally distributed continuous variables. Multivariate logistic regression analysis was performed to determine the impact of the factors on postoperative ESD complications. The regression model included the following factors: age, gender, anxiety, depression, pylori infection, type of lesion, and lesion location. Variables reaching significance, or borderline significance, on univariate analysis (*p* < 0.1) were subsequently incorporated into a multivariate model. In all tests, a *p* < 0.05 was considered statistically significant.

## Result

### Study population

**Figure 2:**
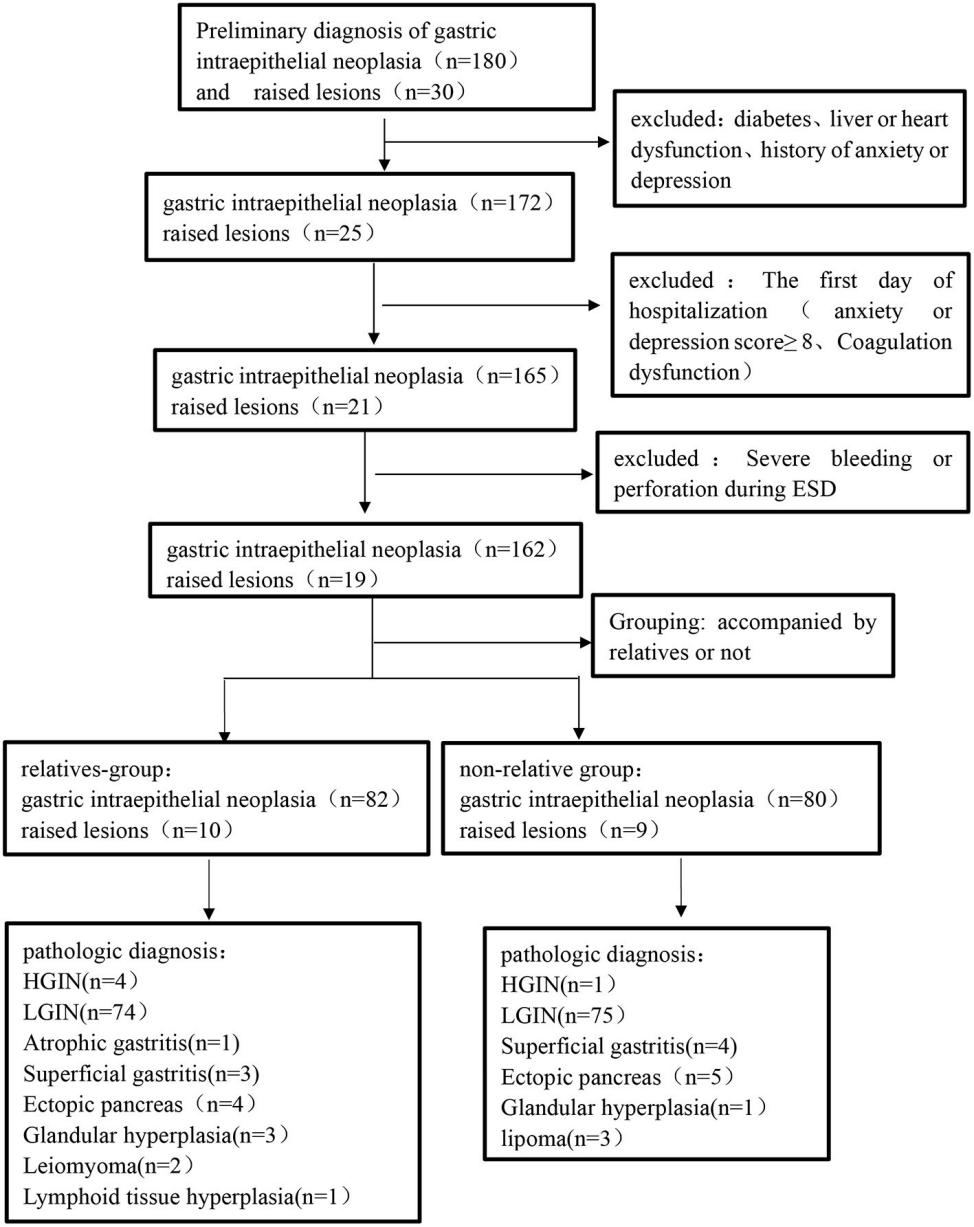
Flowchart demonstrating development of relatives-group and non-relative group.

A total of 180 patients with GI neoplasia and 30 patients with gastric raised lesions were enrolled in our cohort. Among these patients, 24 patients meeting the exclusion criteria and five patients with serious complications during gastric ESD were excluded. Therefore, there were 162 patients with GI neoplasia and 19 patients with a gastric raised lesion in the final analysis. The surgical specimens of each patient were finally diagnosed by pathology (Figure 2). Table 2 lists the demographic and clinical characteristics of the patients.

**Table 2:** Baseline demographics.

**Demographic and clinical variables**	**Relative care group (*n* = 92)**	**Non-relative care group (*n* = 89)**	***p*-value**
Age M (SD)	51.9 (9.1)	50.1 (7.5)	0.15
Age quartiles ≤ 45	24 (26.1%)	29 (32.6%)	0.34
46–53	23 (25.0%)	31 (34.8%)	0.15
54–60	24 (26.1%)	19 (21.3%)	0.45
≥61	21 (22.8%)	10 (11.2%)	0.04
Sex (% female)	49 (53.2%)	41 (46.1%)	0.33
**Lesion location**			
Gastric fundus	21 (22.8%)	15 (16.9%)	0.31
Gastric antrum	34 (37.0%)	45 (50.6%)	0.65
Gastric corpus	20 (21.7%)	15 (16.9%)	0.41
Gastric angle	16 (17.4%)	13 (14.6%)	0.63
Gastric cardia	1 (1.1%)	1 (1.1%)	>0.99
**Type of lesion**			0.76
Intraepithelial neoplasia	78 (84.8%)	76 (85.4%)	0.91
Low-grade	74 (94.5%)	75 (98.7%)	0.37
High-grade	4 (5.1%)	1 (1.3%)	0.37
Atrophic gastritis	1 (1.1%)	0 (0%)	>0.99
Superficial gastritis	3 (3.3%)	4 (4.5%)	0.72
Raised lesions	10 (10.9%)	9 (10.1%)	0.87
Pylori infection	12(13.0%)	13(14.8%)	0.74

### Comparison between groups: Difference in HADS scores

**Figure 3:**
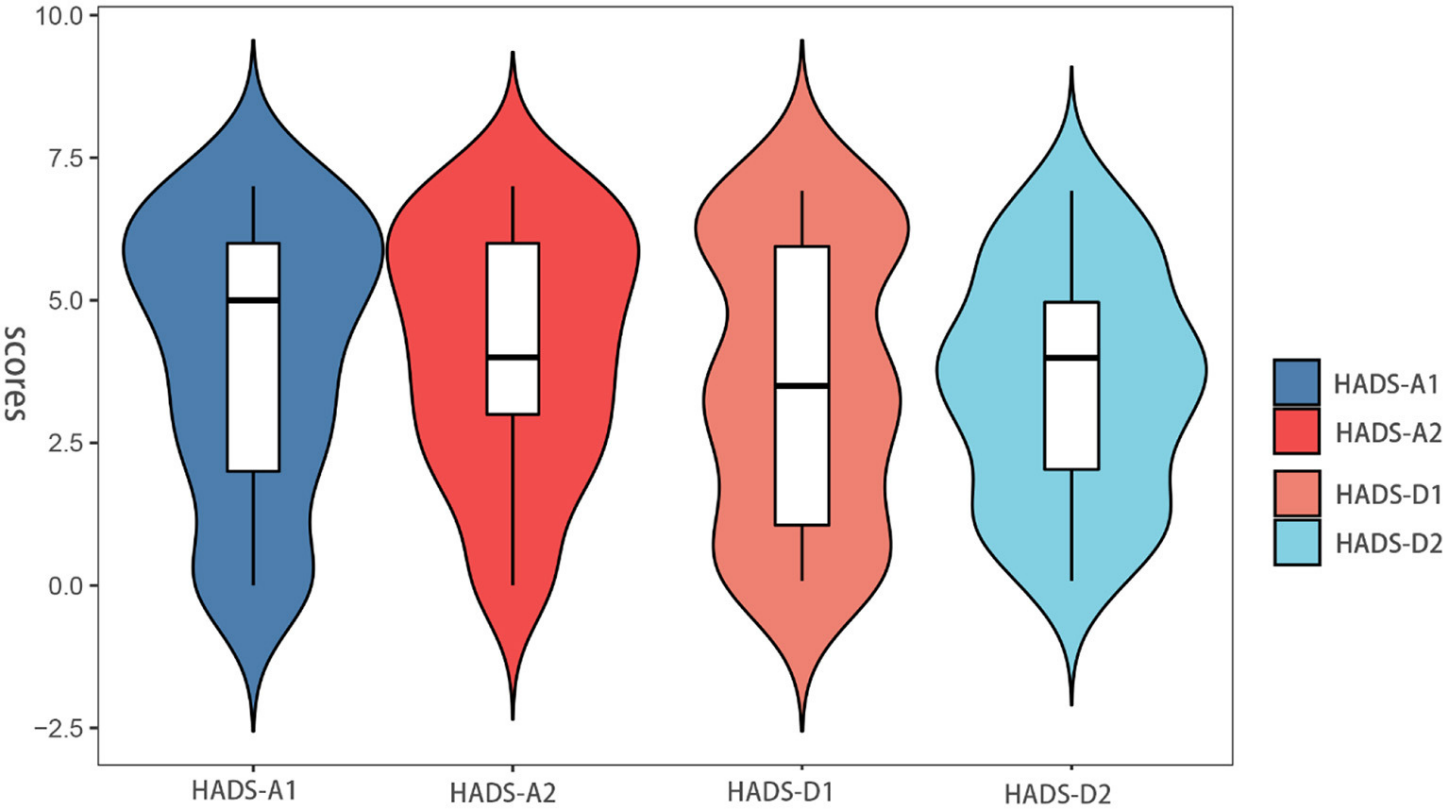
Anxiety and depression scores between pre-ESD of the patient in the relative and non-relative groups.

The anxiety and depression scores of all patients included in this study were ≤ 7 before gastric ESD. The baseline of HADS-A (4.04 ± 2.34 vs. 4.13 ± 2.13, *p* = 0.927) and HADS-D (3.58 ± 2.47 vs. 3.48 ± 2.02, *p* = 0.801) scores was similar between the two groups (Table 3). Figure 3 shows the distribution of differences. However, in terms of postoperative scores, the mean values of HADS-A (4.61 ± 2.89 vs. 5.56 ± 3.07, *p* = 0.042) and HADS-D (4.14 ± 3.03 vs. 4.97 ± 2.61, *p* = 0.048) scores were significantly lower in the relative group compared with the non-relative group (Table 3).

**Table 3:** Difference in anxiety and depression scores between pre-ESD and post-ESD of the patient in the relatives and non-relative groups.

	**Relative care groups**	**Non-relative care groups**	***P*-value**
**Pre-ESD**			
HADS-A	4.04 ± 2.34	4.13 ± 2.13	0.927
HADS-D	3.58 ± 2.47	3.48 ± 2.02	0.801
**Post-ESD**			
HADS-A	4.61 ± 2.89	5.56 ± 3.07	0.042
HADS-D	4.14 ± 3.03	4.97 ± 2.61	0.048
**Post-ESD complication**			
Abdominal pain	8 (8.7%)	17 (19.1%)	0.04
Bleeding	3(3.2%)	3 (3.3%)	>0.99

### Self-contrast: Difference in HADS scores between pre-ESD and post-ESD

**Figure 4:**
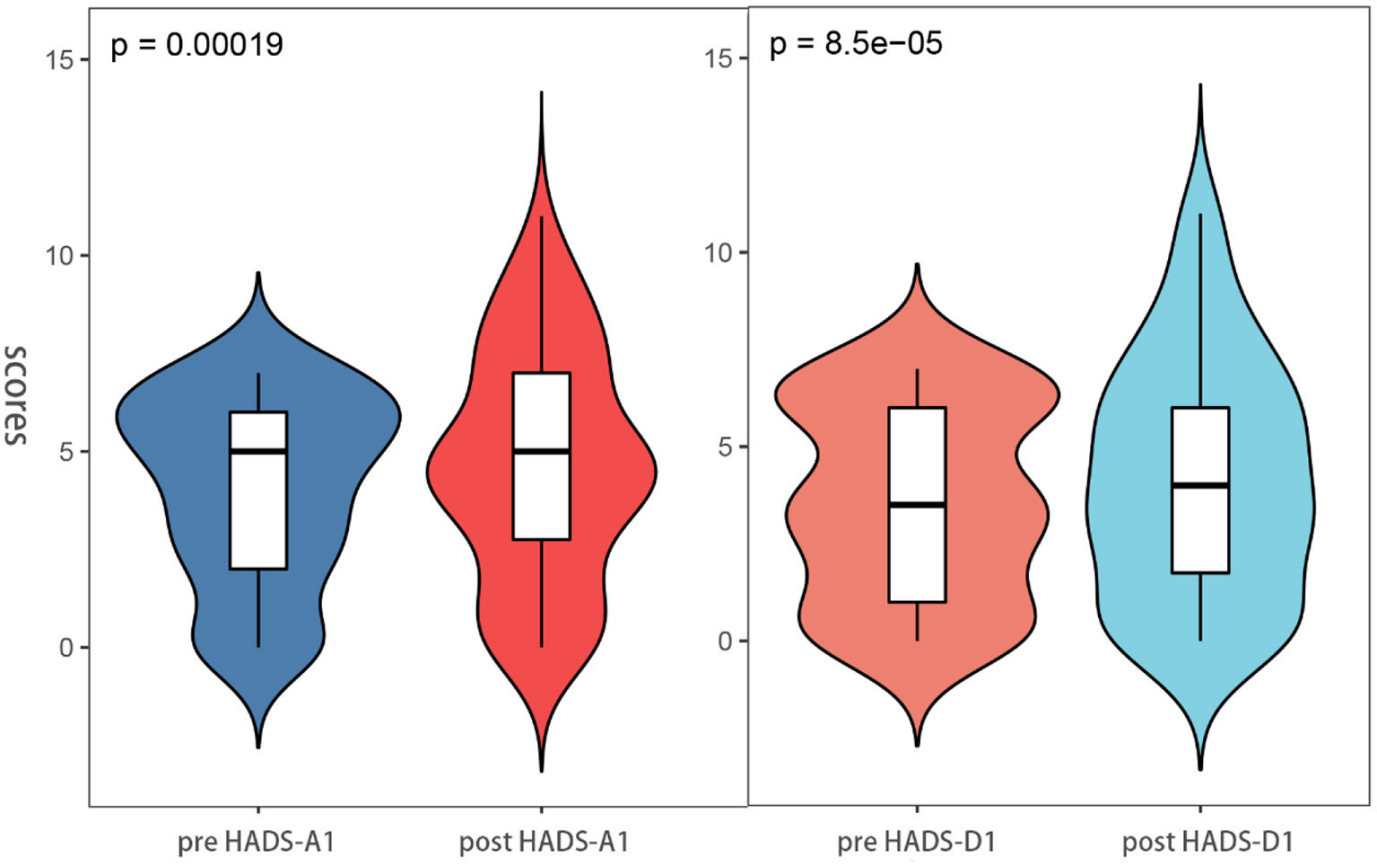
Anxiety and depression scores between post-ESD and pre-ESD of the patient in the relatives groups.

**Figure 5:**
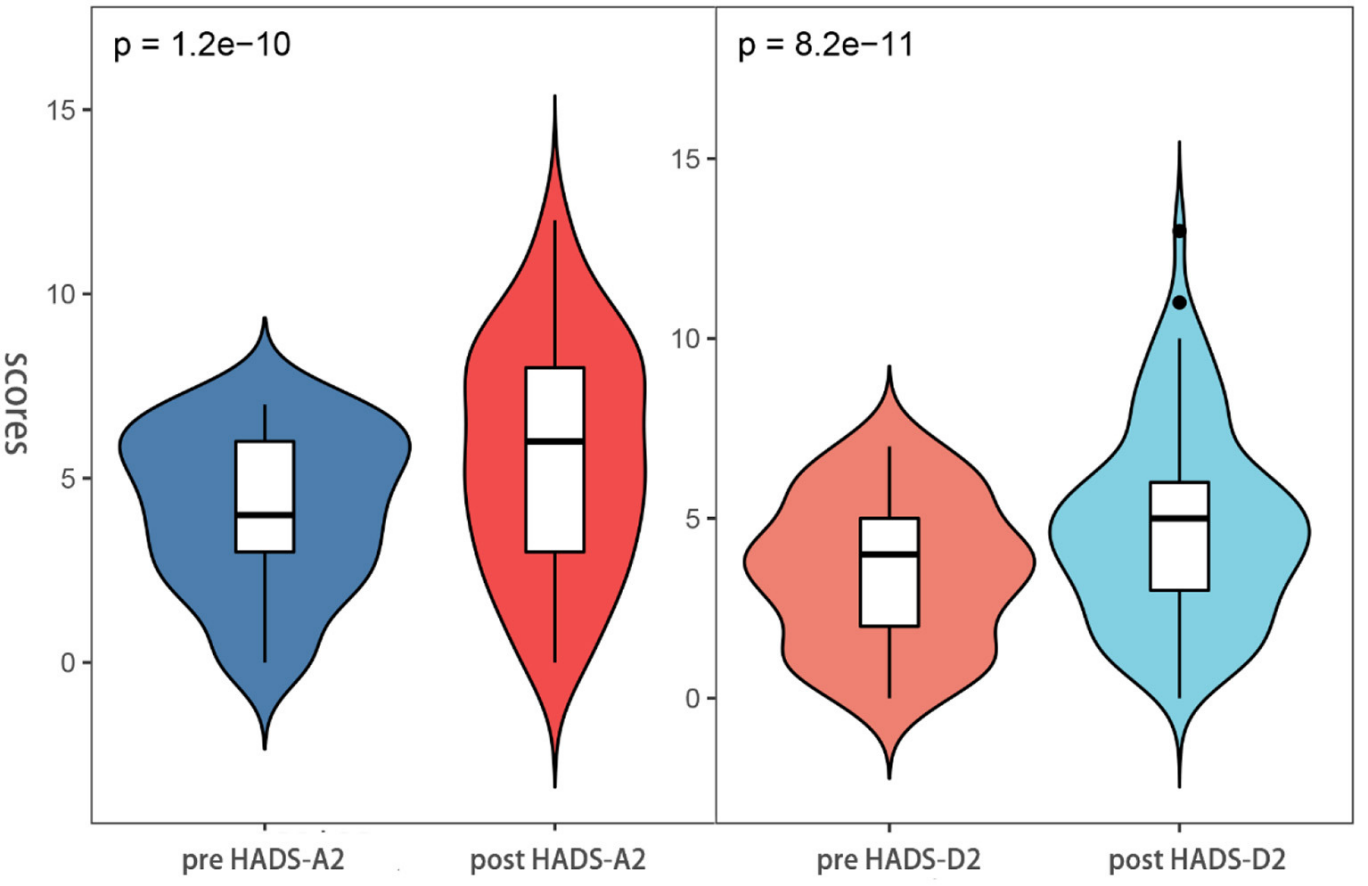
Anxiety and depression scores between pre-ESD and post-ESD of the patient in the non-relatives groups.

Besides, through the pre-ESD and post-ESD self-contrast, the scores of anxiety and depression in the relative group were 0.57 and 0.56, respectively (*p* < 0.001), while those in the non-relative group were increased by 1.43 and 1.49, respectively (*p* < 0.001; Table 4). Figures 4, 5 illustrate the distribution of differences.

**Table 4:** Difference in anxiety and depression scores between post-ESD and pre-ESD of the patient in the relatives and non-relative groups.

	**HADS-A**				**HADS-D**			
	**Pre-ESD**	**Post-ESD**	**d**	***P*-value**	**Pre-ESD**	**Post-ESD**	**d**	***P*-value**
Relatives groups	4.04 ± 2.34	4.61 ± 2.89	0.57 ± 1.63	< 0.001	3.58 ± 2.47	4.14 ± 3.03	0.56 ± 1.27	< 0.001
Non-relative groups	4.13 ± 2.13	5.56 ± 3.07	1.43 ± 1.76	< 0.001	3.48 ± 2.02	4.97 ± 2.61	1.49 ± 1.25	< 0.001

### Risk of complications following gastric ESD

**Figure 6:**
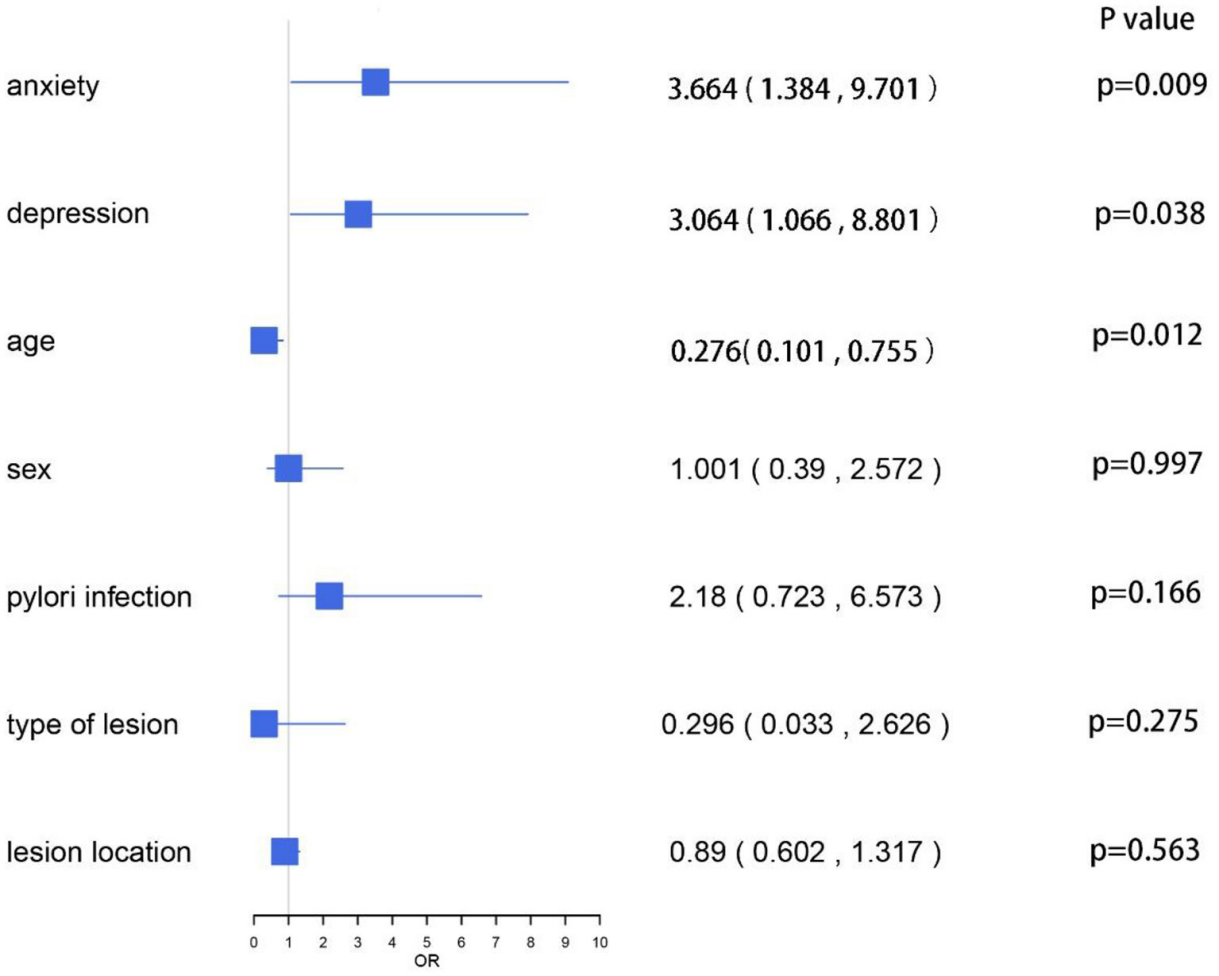
Logistic regression analysis.

After gastric ESD, we observed two types of complications as follows: abdominal pain and bleeding. Table 5 shows the scores of HADS and the incidence of complications in each group. The incidence of anxiety score ≥8 in the non-relative group was higher than that in the relative group (31.5 vs. 18.5%, *P* < 0.05), but there was no significant difference in the incidence of depression score ≥8 between the two groups (14.2 vs. 16.8%, *P* > 0.05). Apparently, the incidence of abdominal pain in the non-relative group was significantly higher compared with the relative group (19.1 vs. 8.7%, *p* = 0.04). However, there was no significant difference in the incidence of bleeding between the two groups. We conducted multivariate logistic regression analysis for the complications. The results showed that HADS-A scores ≥8 (OR, 3.664; 95% CI, 1.384 ~ 9.701, *P* = 0.009), HADS-D scores ≥ 8 (OR, 3.064; 95% CI, 1.066 ~ 8.801, *P* = 0.038) and individuals aged < 45 years (OR, 0.276; 95% CI, 0.101 ~ 0.755, *P* = 0.012) were significantly associated with post-ESD complications. Other factors, such as sex, H. pylori infection, type of lesion, and lesion location, were not associated with post-ESD complications (*p* > 0.05; Figure 6).

**Table 5:** post-ESD complication and HADS scores.

	**Relatives groups (*n* = 92)**	**Non-relative groups (*n* = 89)**	***P*-value**
**HADS-A**			
≤ 7	75 (81.5%)	61 (68.5%)	0.04
8~10	16 (17.4%)	24 (27.0%)	0.12
≥11	1 (1.1%)	4 (4.5%)	0.20
**HADS-D**			
≤ 7	79 (85.9%)	74 (83.1%)	0.61
8~10	10 (10.9%)	13 (14.6%)	0.45
≥11	3 (3.3%)	2 (2.2%)	>0.99
**Post-ESD complication**			
Abdominal pain	8 (8.7%)	17 (19.1%)	0.04
Bleeding	3 (3.3%)	3 (3.4%)	>0.99

## Discussion

As one of the most studied areas of psychosomatic diseases, the relationship between negative emotions and peptic ulcers has been intensively investigated (26, 27). Similarly, negative emotions can also affect the healing of iatrogenic ulcers caused by gastric ESD. Overall, the prevalence of psychological distress in this study was about 19%, which was relatively lower compared with previous studies on patients with gastric lesions in other countries (28, 29). Such discrepancy might be attributed to the fact that the included patients in previous studies are diagnosed with different stages of gastric cancer before evaluation. Instead, we recruited only patients with dysplasia and raised lesions. Besides, on the following day of admission, patients with an abnormal value of HADS scores were excluded. When patients know that they have gastrointestinal lesions and need gastric ESD surgery, they first feel that they are on the verge of death. Because of fear of unknown diseases and operation, they may be more likely to develop psychological disorders during the perioperative period (30, 31). Some studies have pointed out that 22–58% of patients with malignant tumor have depression, anxiety and other psychological disorders (29, 32). Moreover, the suicidal tendency of these patients was 2–3 times higher than that of the general population (33).

When patients know that they need to do gastric ESD, they may be prone to psychological disorders during the perioperative period due to apprehension about unknown diseases and fear of surgery. In our observational study, we found that the HADS scores of all patients were increased in varying degrees. However, such an increase in patients in the non-relative group was more obvious. Besides, we found that the average anxiety and depression scores of the relative group were lower compared with the non-relative group (*p* < 0.05). Some studies (34) have shown that patients who need gastric ESD to treat early gastric cancer were given systematic psychological intervention, and the anxiety and depression scores of patients after intervention were significantly lower than those before. However, in this study, the anxiety and depression scores increased after ESD, which may be due to some reasons: First, we recorded HADS scores on the first day after operation, when the pathological results were not yet available, which made the patient feel uneasy. Second: the intervention measures of this study are family care, while other studies are systematic psychological intervention, which include cognitive or behavioral therapies, integrative therapy, family therapy, psychodynamic therapy, humanistic therapy, interpersonal psychotherapy, and non-directive therapy (35, 36). Our finding indicated that for hospitalized patients, the accompanying of relatives could reduce the occurrence and degree of anxiety and depression to a certain extent. They could help patients with psychological counseling. Therefore, the patients could maintain a relatively positive and optimistic attitude, leading to reduced impact of psychological factors on the disease. In addition, some studies have shown that the occurrence of bad emotions is negatively correlated with family support (34, 37). Psychotherapy under the guidance of relatives can enable patients to master the relevant knowledge of gastrointestinal diseases and improve the behavior of following doctors' orders, which is similar to the health education in the relevant literature (38, 39).

Routine administration of PPI can suppress gastric acid secretion and promote ulcer healing after ESD (40–42), resulting in retarded development of post-ESD bleeding. The occurrence of anxiety, depression and other bad emotions may come from various complications after ESD, such as abdominal pain, perforation, bleeding and so on Zhao and Wang (43). In terms of complications after gastric ESD, we found six cases of bleeding and 25 cases of abdominal pain. In the relative group, abdominal pain occurred in 8 cases (8.7%) and bleeding in 3 cases (3.3%). In the non-relative group, abdominal pain occurred in 17 cases (19.1%) and bleeding in 3 cases (3.4%). A meta-analysis including 11 studies showed that the incidence of postoperative bleeding after ESD in early gastric cancer was about 6.4%. Due to the low incidence of gastric bleeding after ESD and the relatively insufficient sample size in this study, there was no significant difference in the incidence of gastric bleeding after ESD between the two groups. However, in terms of abdominal pain after ESD, the incidence of abdominal pain in the non-relative group was significantly higher than that in the relative group (19.1 vs. 8.7%, *P* < 0.05). At the same time, the incidence of anxiety score ≥8 in the non-relative group was also significantly higher than that in the relative group (31.5 vs. 18.5%, *P* < 0.05), suggesting that there may be a positive correlation between abdominal pain and anxiety. Relatively speaking, patients in the non-relative group were more likely to undergo complications (*p* < 0.05). By multivariate logistic regression analysis, we found that the ESD complications showed a positive correlation with all subscales of HADS scores and age, while they were not associated with lesion location, type, H. pylori infection, and gender. In conclusion, the relative group had a lower HADS score and a lower incidence of post-ESD complications.

During the perioperative period, we should try our best to persuade family members to take care of patients, especially elderly patients. Through strengthening humanistic care, the production of bad emotions could be reduced to promote the healing of ESD-induced ulcer. Actively relieving psychological pressure in the process of family care is an effective measure to reduce anxiety, depression and postoperative complications after gastric ESD. At the same time, providing a clean, warm and comfortable hospital ward environment and establishing scientific and appropriate rest time and diet management is also a key step. In such an environment, patients' mood will appear relaxed, and high-quality sleep time can also enable patients to keep an optimistic attitude (44).

This observational study has several obvious limitations. First, this was a single-center cross-sectional study with a relatively small sample size, which might limit the reliability of the results. Second, patients with a previous history of mental disorders and an admission HADS score ≥8 were excluded. Therefore, the impact on these patients remained unknown. Third, instead of classifying patients based on the type and location of gastric lesions, we evaluated all patients together.

## Conclusion

Our study revealed that patients receiving gastric ESD under the care of relatives during hospitalization had lower HADS-A and HADS-D scores compared with the non-relative group. Besides, the incidence of post-ESD abdominal pain in the relative group was significantly lower compared with the non-relative group. These findings suggested that patients receiving gastric ESD who were accompanied by their families were more conducive to emotional stability, may showing less postoperative clinical manifestations.

## Data availability statement

The original contributions presented in the study are included in the article/supplementary material, further inquiries can be directed to the corresponding authors.

## Author contributions

R-YGao, R-YGan, and J-LH prepared the tables and drafted the manuscript. B-HW and T-TL reviewed the manuscript for its intellectual content. L-SW, D-FL, and JY were responsible for revising the manuscript. All authors have read and approved the final manuscript.

## Funding

JY is supported by the National Natural Science Foundation of China (No. 81800489). L-SW is supported by the Technical Research and Development Project of Shenzhen No. JCYJ20170307100911479. D-FL is supported by the Natural Science Foundation of the Guangdong Province (No. 2018A0303100024).

## Conflict of interest

The authors declare that the research was conducted in the absence of any commercial or financial relationships that could be construed as a potential conflict of interest.

## Publisher's note

All claims expressed in this article are solely those of the authors and do not necessarily represent those of their affiliated organizations, or those of the publisher, the editors and the reviewers. Any product that may be evaluated in this article, or claim that may be made by its manufacturer, is not guaranteed or endorsed by the publisher.
